# Supplementary material for: Effectors and potential targets selectively upregulated in human KRAS-mutant lung adenocarcinomas
Source: Sci Rep. 2016 Jun 15;6:27891. doi: 10.1038/srep27891 (PMC4908391; doi:10.1038/srep27891)
Supplement: Supplementary Information [file srep27891-s1.pdf]

Effectors and potential targets selectively upregulated in human *KRAS*-mutant lung adenocarcinomas

Jinyu Li[1], Raffaella Sordella[2], and Scott Powers[1,2,3]

Supplementary Information

Supplementary Table Legends

Supplementary Table 1. Protein levels in three groups of lung adenocarcinomas**.** Mean levels in three groups are listed along with the p-values and effect sizes of pairwise comparisons. G1 = *KRAS*-mutants; G2 = other Raf/MAPK pathway mutants; G3 = all others.

Supplementary Table 2. Relative protein levels in four groups of *KRAS*-wild-type lung adenocarcinomas that have other mutations in the RTK/RAF/MAPK pathway. p-values in the four groups are listed along with the effect sizes of pairwise comparisons.

Supplementary Table 3. Protein levels in three groups of *KRAS*-mutant lung adenocarcinomas**.** Mean levels in three groups are listed along with the p-values and effect sizes of pairwise comparisons. G1 = *KRAS/TP53* double mutants; G2 = *KRAS/STK11* or *KRAS/KEAP1* double mutants and *KRAS/STK11/KEAP1* triple mutants; G3 = all others.

Supplementary Table 4. RNA levels in three groups of lung adenocarcinomas**.** Mean levels in three groups are listed along with the p-values and effect sizes of pairwise comparisons. G1 = *KRAS*-mutants; G2 = other Raf/MAPK pathway mutants; G3 = all others.
